# The Forgotten Ones: Crafting for Meaning and for Affiliation in the Context of Finnish and Japanese Employees' Off-Job Lives

**DOI:** 10.3389/fpsyg.2021.682479

**Published:** 2021-08-30

**Authors:** Miika Kujanpää, Oliver Weigelt, Akihito Shimazu, Hiroyuki Toyama, Merly Kosenkranius, Philipp Kerksieck, Jessica de Bloom

**Affiliations:** ^1^Faculty of Social Sciences (Psychology), Tampere University, Tampere, Finland; ^2^School of Business, University of South-Eastern Norway, Hønefoss, Norway; ^3^Institute of Psychology Wilhelm Wundt, Leipzig University, Leipzig, Germany; ^4^Department of Policy Management, Keio University, Tokyo, Japan; ^5^Faculty of Educational Sciences, University of Helsinki, Helsinki, Finland; ^6^Faculty of Economics & Business, University of Groningen, Groningen, Netherlands; ^7^Epidemiology, Biostatistics and Prevention Institute, University of Zurich, Zurich, Switzerland

**Keywords:** off-job crafting, context of crafting, meaning, affiliation, vitality, recovery experiences, DRAMMA model

## Abstract

In an intensifying working life, it is important for employees to proactively shape their lives beyond work to create opportunities for satisfying personal needs. These efforts can be beneficial for creating and sustaining well-being in terms of vitality. In this study, we focused on off-job crafting (OJC) for meaning and OJC for affiliation, conceptualized as proactive changes in off-job life with the aim of increasing satisfaction of needs for meaning and affiliation, among employees in Finland and Japan, two countries with disparate cultural values. We examined longitudinal within-person relationships between the two OJC dimensions and vitality, as well as the relationships between OJC and contextual variables, such as age and gender. We conducted a longitudinal study over 6 months with three measurement points. A total of 578 Finnish and 228 Japanese employees participated in the study. Hypotheses were tested with latent growth analysis. Increases in OJC for meaning and for affiliation were mostly positively related to increases in vitality over time in both countries. In Finland, age was positively related to OJC for meaning. In Japan, age was negatively related to OJC for meaning, but the female gender was positively related to OJC for affiliation. Focusing on increasing meaning and affiliation in off-job life can be beneficial strategies for employees to feel positively energized. The role of contextual variables and culture in OJC should be examined further in future studies.

## Introduction

“*Happiness is thought to depend on leisure; for we are busy that we may have leisure*” (Aristotle, 2009, p. 195).

How can one craft a life worth living? How can one proactively shape his/her life to experience well-being and to feel energized and alive? Modern working life exposes employees to all sorts of busyness in the form of intensifying job tasks and high workload (Rosa, 2013; Kubicek and Tement, 2016), with little time or guidance for identifying aspects of the various activities that are truly important on a personal level. Leisure plays a central role in balancing the challenging work domain and in fostering health, well-being, and a sustainable working life (e.g., Kuykendall et al., 2015; Zawadzki et al., 2015). Numerous studies have shown that the four recovery experiences of detachment from work, relaxation, control, and mastery in leisure time measured with the recovery experience questionnaire (REQ; Sonnentag and Fritz, 2007) are closely connected to mental well-being and energy (Sonnentag et al., 2017; Bennett et al., 2018; Steed et al., 2021). However, these studies in the field of recovery from work have been driven by a rather narrow conceptualization of off-job life as the absence of stress and work tasks (Eden, 1990; Meijman and Mulder, 1998). Integrating knowledge from leisure sciences and the wider well-being benefits of leisure with recovery research can complement and go beyond this earlier perspective (Stebbins, 2015; Kelly et al., 2020).

The DRAMMA (i.e., detachment, relaxation, autonomy, mastery, meaning, and affiliation) model addresses these objectives and integrates insights from the fields of recovery from work and leisure sciences (Newman et al., 2014; Kujanpää et al., 2020). Based on a literature review of 363 scientific articles, Newman et al. (2014) proposed that six psychological experiences mediate the positive relationship between leisure and well-being. In addition to the recovery experiences of detachment from work, relaxation, autonomy (originally referred to as “control” in recovery research), and mastery, which had previously been identified and investigated intensively (Sonnentag and Fritz, 2007; for metaanalyses, see Bennett et al., 2018; Steed et al., 2021), two important factors of recovery from work were newly added in the DRAMMA model, which are *meaning and affiliation*. These two experiences, which are “forgotten ones” in recovery research will be the focus of this study, more specifically, *proactive efforts* of employees to experience meaning and affiliation through their off-job life, their role in fostering vitality, and their contextual antecedents (i.e., age, gender, human capital, and working hours).

### Crafting for Meaning and for Affiliation

Research to date has shown that some meaning- and affiliation-related experiences (the latter also interchangeably referred to as “relatedness” or “belongingness” in the literature) and activities, such as engagement in personally meaningful activities (Hooker et al., 2020), experiences of meaning in life (Hadden and Smith, 2019), meaningfulness (Crego et al., 2020), as well as sharing positive experiences with others (Lambert et al., 2011), and feelings of relatedness (Martela et al., 2016) can provide a source of enhanced vitality, conceptualized as “the subjective experience of being full of energy and alive” (Bostic et al., 2000, p. 313). Some studies have also reported inconsistent or mixed results. For instance, strategies for finding a purpose in life turned out to be negatively related to well-being (e.g., search for meaning; Li et al., 2020; struggle with ultimate meaning; Wilt et al., 2018) or have shown both positive and negative relationships to different aspects of well-being (meaning-making; Park, 2010). In addition, research on the role of meaning and affiliation as enhancers of the recovery process beyond recovery experiences of the REQ has rendered both null and positive effects (Kujanpää et al., 2020; Virtanen et al., 2020). As meaning and affiliation at leisure time are highly culturally- and contextually-shaped experiences (Iwasaki, 2018), examining these two newer facets of recovery experiences in distinct cultural contexts is meaningful.

Importantly, we suggest that the mixed findings so far could originate from the conceptualization of meaning and affiliation in the body of research on recovery experiences and the DRAMMA model as *passive experiences* that happen by coincidence rather than experiences that can be *proactively shaped* (i.e., *crafted*; de Bloom et al., 2020). As such, the active role that the employees can play in striving for and making these experiences happen has been overlooked. We propose and investigate a novel perspective that employees can proactively craft off-job experiences of meaning and affiliation, which in turn is expected to enhance their vitality, an indicator of well-being (Ryan and Frederick, 1997). We test these propositions empirically in a longitudinal survey among Finnish and Japanese employees. We focus on two proactive off-job crafting (OJC) strategies, which are OJC for meaning and for affiliation as potential enhancers of vitality. Moreover, we examine similarities and differences in the relationship between OJC and contextual antecedents among Finnish and Japanese employees. OJC for meaning and for affiliation are conceptualized as proactive and self-initiated changes in the off-job life of an employee (including off-job life domains such as leisure, hobbies, voluntary work, and child- and housecare) that aimed at increasing satisfaction of the need for meaning/affiliation (Kujanpää et al. manuscript under review).

### Contributions and Research Aims

This study makes four key contributions to the literature. First, studying if and how experiences of meaning and affiliation can be proactively shaped and crafted for. advances the literature on recovery experiences by focusing on these two recovery experiences newly added in the DRAMMA model (Newman et al., 2014), which have previously been neglected in recovery research. Second, the concept of OJC contributes to the recovery literature by adding the perspective of recovery as a proactive process. Third, we use an advanced longitudinal measurement design with three measurement occasions, with an analogous measurement approach in Finland and Japan to examine the relationship between OJC and vitality. Thus, our study follows recent calls in crafting research to examine within-person changes across time (e.g., Rofcanin et al., 2019). Fourth, we investigate crosscultural differences in OJC in the two countries that differ widely as regards cultural values and the perceived value of leisure as compared to work (i.e., Finland and Japan; Hofstede et al., 2010). Examining the promoting or inhibiting role of contextual variables (e.g., age, gender) for crafting in distinct cultures is important to understand how often and in which ways people engage in crafting (see also Zhang and Parker, 2019). Thus, we provide novel insights on the role of the cultural context for OJC.

### OJC as a Strategy to Enhance Vitality

By proactively shaping off-job life, employees can match their off-job activities with their personal needs, goals, and interests (Berg et al., 2010; Demerouti et al., 2020). The concept of OJC was recently developed based on the DRAMMA model (Kujanpää et al. manuscript under review). The dimensions of (1) OJC for meaning and (2) OJC for affiliation, respectively, refer to proactively shaping the off-job life a person to (1) provide a sense of purpose and (2) allow for more opportunities for experiencing a sense of close connectedness with relevance to others.

Earlier research has supported the notion that proactive crafting efforts in the off-job domain have positive implications for experiencing meaning and affiliation. Petrou and Baker found that weekly crafting of leisure time related positively to weekly relatedness satisfaction (Petrou and Bakker, 2016), as well as to meaning-making (Petrou et al., 2017). However, we know very little about the role OJC plays for other important indicators of well-being, such as vitality. For the purposes of this study, we chose vitality as the examined outcome since it is closely related to both hedonic (seeking pleasure) and eudaimonic (seeking to develop oneself) well-being (Huta and Ryan, 2010).

The integrative needs model of crafting (de Bloom et al., 2020) posits that OJC efforts lead to higher well-being through the satisfaction of psychological needs, such as meaning and relatedness. Thus, experiences of meaning and affiliation gained through successful OJC efforts can serve as personal resources that generate well-being and optimal functioning over time (de Bloom et al., 2020). In accordance with this idea, and based on the model of human energy by Quinn et al. (2012), we propose that satisfaction of meaning and affiliation needs, experienced through successful OJC, accumulates the supply of personal resources, and increases energetic activation (vitality) over time across different cultural contexts. Thus, we expect that (1) OJC for meaning and (2) OJC for affiliation, i.e., proactively shaping off-job life with the goal of increasing the satisfaction of the need for (1) meaning and (2) affiliation, are both positively related to vitality among both Finnish (H1) and Japanese employees (H2) longitudinally at the within-person level. We examined within-person effects to focus on the individual, state-like variation over time in OJC and vitality (Ilies et al., 2015; McCormick et al., 2020).

### OJC in Context

In addition to studying the relationship between OJC and vitality in two culturally distinct countries, we also examined the role of cultural and demographic factors in how often people engage in OJC for meaning and for affiliation. We argue that examining the possible cultural mechanisms that interplay with demographic variables helps to create a more refined picture of the conditions that increase or decrease the crafting efforts of people (see also Urbach et al., 2020). Despite calls for crafting research in different cultural contexts, studies adopting a crosscultural framework are scarce (Erez, 2010; Schachler et al., 2019; Zhang and Parker, 2019). Moreover, although demographic variables such as age and gender are routinely controlled for in crafting studies (e.g., Bindl et al., 2019), their role in crafting has been only rarely examined (for a notable exception, see Kooij et al., 2017). We focus on OJC in a Western and an Eastern country, Finland and Japan, respectively. While both are developed, are high-income, and industrialized countries, they are very different in terms of access to leisure time, the perceived value of leisure compared with that of work, as well as in cultural values, especially for the value dimensions of long-/short-term orientation and masculinity–femininity (Hofstede et al., 2010). This makes these countries interesting examples to examine how crafting is influenced by dissimilar cultural contexts. For instance, Finland is a state with a strong social welfare regime, with strictly enforced regulations on employee working hours and social security (Virtanen et al., 2018). On the other hand, there are far fewer labor regulations in Japan, and employees tend to prioritize their careers more often above the leisure and home domains (Peltokorpi, 2013; Isakjee, 2017).

Most crafting research to date has been conducted in Western, individualistic countries (Sakuraya et al., 2017). Comparing crafting antecedents, efforts and outcomes across both a Western (i.e., Finland) and an Eastern country (i.e., Japan) will render new insights on the extent to which OJC as a proactive approach to shaping off-job life is universally applicable or dependent on the cultural context (see also Lomas, 2015). To learn about the effects of individual-level demographic and employment characteristics on OJC, we chose to focus on four commonly assessed variables which are highly relevant in an occupational context (Rudolph et al., 2017), namely, age, gender, human capital (education and tenure), and working hours.

As people age, they tend to internalize the values of their cultural context (Fung, 2013). Since motives related to seeking emotional meaning and social contact increase in prominence globally as people age (Carstensen et al., 1999), we expect that chronological age is positively related to OJC for meaning and for affiliation in both Finland (H3a) and in Japan (H3b). Moreover, we propose that the cultural value dimension of long-/short-term orientation may be relevant with regard to age. In short-term orientation focused countries, such as Finland, the focus of the outcomes of organizational and personal activities is on the immediate future. Countries with a high long-term orientation, such as Japan, which has one of the highest values in the world on this dimension, are characterized by focusing on the importance of long-term gains (Hofstede et al., 2010). Accordingly, while the Finnish media portray the aging workforce with negative connotations, the Japanese media describe aging in more positive terms, emphasizing the value and knowledge aging employees contribute to society (Ishikawa Unpublished dissertation). Future-oriented planning promotes proactive and goal-oriented behavior, such as crafting (Grant and Ashford, 2008; Parker et al., 2010). Due to the pronounced long-term orientation focus in Japan (Hofstede et al., 2010), we expect that the positive relationships between age and OJC for meaning, and age and OJC for affiliation are stronger in Japan than in Finland (H3c).

Finland and Japan differ markedly in the extent to which gender roles affect how people live their lives. These differences can be explained by the cultural value dimension of masculinity–femininity, defined as the valuation of earnings, recognition, advancement, and challenges (masculine values) vs. valuation of relationships, cooperation, and security (feminine values) (Hofstede et al., 2010). While Finland is quite a feminine culture, Japan is one of the most masculine cultures in the world (Hofstede et al., 2010). Accordingly, Finnish men and women spend fairly equal time at work (Lee et al., 2007). Due to relatively equal gender norms regarding leisure and housecare in Finland, we expect that gender will not be strongly related to OJC in Finland. In Japan, traditional gender roles play a greater role in that women are often expected to take care of housecare (Osawa, 2020). Furthermore, in spite of recent trends toward more gender equality in Japanese organizations (e.g., Kurokawa, 2020; Shimazu, 2020), male Japanese employees quite often prioritize their careers over their off-job lives and have more opportunities to do so than female employees (Nemoto, 2013; Usui, 2018). While female Japanese employees may be able to shape their off-job lives by crafting for meaning and for affiliation, male Japanese employees may have fewer opportunities for OJC and may prioritize work over other activities (Peltokorpi, 2013). Thus, we expect that gender is more strongly related to OJC in Japan (than in Finland), implying that female Japanese employees are expected to engage in OJC more than male employees (H4).

Human capital refers to the skills and knowledge the employees had gained through education and training, which increases the chances of success in the job market (Becker, 1964). It is commonly operationalized as education and tenure (e.g., Ng and Feldman, 2010). Human capital helps employees to self-manage their careers and work–home boundaries (Sturges, 2008). As education and tenure can produce skills that translate to the off-job domain (e.g., Wilhelm and Hirschi, 2019), they make OJC efforts potentially more likely and efficient. Moreover, experiencing successes through skillful crafting efforts may promote further crafting (de Bloom et al., 2020). Accordingly, we expect that human capital (education and organizational tenure) is positively related to OJC for meaning and for affiliation among both Finnish (H5a) and Japanese (H5b) employees. However, this relationship may also be affected by the cultural working context. Masculine cultures emphasize on a “performance society” over other foci in life (Hofstede et al., 2010). In the masculine Japanese work context, human capital also promotes more pronounced work centrality and embeddedness in work (Ono, 2018). Highly educated and tenured Japanese employees tend to become more firmly embedded in the organization and their professional networks, which makes pursuing non-work-related goals less crucial for them (Peltokorpi, 2013). Thus, we expect that the positive relationships between human capital (education and organizational tenure) and OJC are weaker for Japanese than for Finnish employees (H5c).

In the decidedly masculine Japanese culture, employees are expected to work long hours, with working days often extending to 10–12 h (Nemoto, 2013). Moreover, it is common to spend several hours after the regular working day in more informal meetings with employers or clients (Ikeda et al., 2011), and commuting takes the average Japanese worker almost 1.5 h each day (Statistics Bureau of Japan, 2016), which further decreases their available leisure time. Working very long hours, Japanese employees may need to use their limited off-job time primarily for activities such as personal care and sleep. In Finland, a typical working week lasts 37 h (OECD. Stat Extracts, 2020), and leisure time is generally seen as an important part of life (Hofstede et al., 2010; Wang and Wong, 2014). Consequently, employees in Finland have more leisure time and more opportunities to shape their working hours to match their needs (Härmä, 2006). Working long hours may therefore reduce OJC, especially among Japanese employees, but not as much for Finnish employees. Thus, we expect that there is a negative relationship between working hours and OJC among both Finnish (H6a) and Japanese employees (H6b), and that this link is stronger in Japanese employees (H6c).

## Materials and Methods

### Procedure and Participants

We conducted a longitudinal study with three measurement points between 2018 and 2019 among Finnish (*n* = 578) and Japanese (*n* = 228) employees recruited through various organizations. There were 3-month time lags between measurements, following calls for more “shortitudinal” study designs (Dormann and Van de Ven, 2014; Dormann and Griffin, 2015). All employees had to work at least 24 h per week to be able to participate. The participants provided informed consent. The samples differed between countries. The mean age was 48.70 years (*SD* = 10.23) among Finnish employees and 30.86 years (*SD* = 6.35) among Japanese employees. A total of 85% of Finnish and 37% of Japanese employees were female, and 50% of the Finnish and 95% of the Japanese employees had an academic degree. Less than half (39%) of the Finnish employees and one fourth (24%) of Japanese employees had at least one child living at home. Finnish employees worked on an average of 39 h and Japanese employees 48 h per week (including unpaid overtime). Finnish employees worked mainly in the public sector, such as health care and education, whereas Japanese employees worked mainly in information technology.

### Statistical Analyses

The hypotheses were examined using latent growth analysis (LGA, see Supplementary Material) in Mplus 8.4 (Muthén and Muthén, 1998). LGA is well-suited for examining within-person changes in a predictor variable and outcome over time (McArdle, 2009). In LGA, two latent growth curve parameters are estimated: the intercept (i.e., the initial level) and the slope (i.e., the rate of change over time). Moreover, growth curves for two or more variables can be created to examine the relationships between the intercepts and slopes of those variables (McArdle, 2009). Robust maximum likelihood estimation was used to take into account missing values and potential deviances from normality (Muthén and Muthén, 1998). We used a full information maximum likelihood approach that allowed us to use all the observations in the data to estimate model parameters without imputing data. Following Schermelleh-Engel et al. (2003), we evaluated model fit with commonly used indicators, namely: CFI, TLI, RMSEA, and SRMR. For TLI and CFI, values above 0.90 indicate acceptable fit. For RMSEA, values under 0.05 indicate good model fit, whereas values between 0.05 and 0.08 indicate acceptable fit. For SRMR, values below 0.08 indicate good fit, whereas values between 0.08 and 0.10 indicate acceptable fit (Schermelleh-Engel et al., 2003).

**Figure 1:**
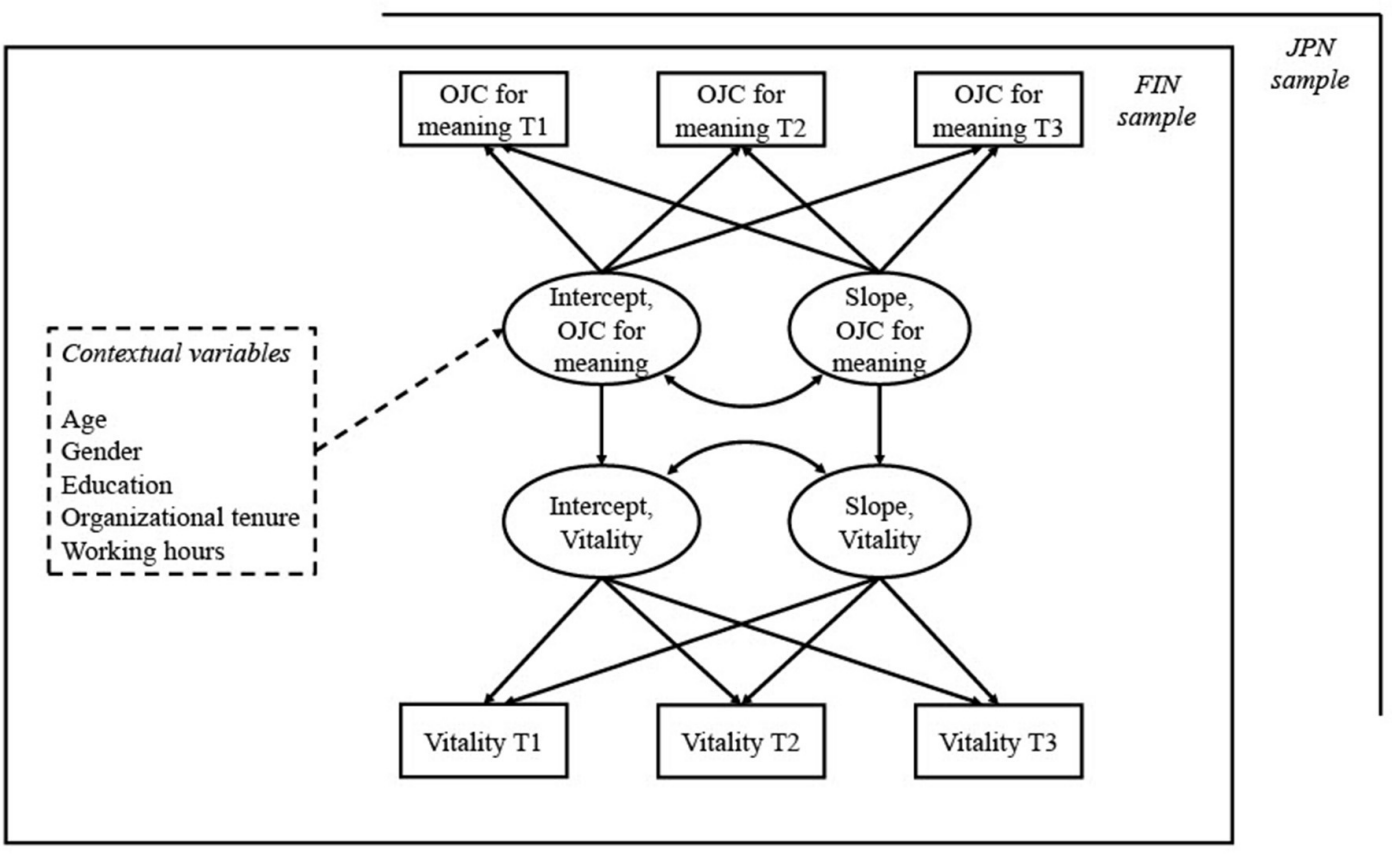
Multivariate growth curve model for OJC for meaning, vitality, and contextual variables. Contextual variables (dashed lines) were added to the model in a second step. A similar model was made separately for OJC for affiliation. OJC, off-job crafting; FIN, Finnish sample; JPN, Japanese sample.

We used multigroup LGA to estimate models simultaneously for the Finnish and Japanese employees. First, univariate latent growth curve models were created for OJC for meaning, OJC for affiliation and vitality to examine stability and development during the 6-month study period in these variables. Next, we estimated bivariate models separately for the relationships between OJC for meaning and vitality, and between OJC for affiliation and vitality. We estimated the relationships between intercepts (i.e., the initial values) as well as the relationships between slopes (i.e., the developments over time) of OJC and vitality. These analyses thus show whether OJC and vitality covary across time (Hypotheses H1–H2). Finally, we added the contextual variables (gender, age, education, organizational tenure, and working hours) as predictors of the intercepts of OJC (Figure 1). Hypotheses H3–H6 were then tested by examining the significance of the contextual variables in predicting the initial values of OJC for meaning and for affiliation. We report standardized estimates throughout all analyses.

### Measures

Chronological age, gender, human capital (education and organizational tenure), and working hours (weekly contractual hours plus hours worked over and above contractual hours) were measured at T1. OJC was measured with the needs-based off-job crafting scale (NOCS; Kujanpää et al. manuscript under review) at all three time points, with three items for both OJC for meaning and for affiliation. Measurement properties demonstrating strong measurement invariance for the NOCS in the Finnish and Japanese sample are reported elsewhere (Kujanpää et al. manuscript under review). All items started with “Over the past month….” Example items are “I've made sure to experience meaning in my life during off-job time” for crafting for meaning and “I've made sure to experience close connections to the people around me during off-job time” for crafting for affiliation. Answers could range from 1 (never) to 5 (very often). Cronbach's alphas from T1 to T3 were 0.88, 0.89, and 0.87 for crafting for meaning and 0.86, 0.87, and 0.90 for crafting for affiliation, respectively in Finland. Cronbach's alphas from T1 to T3 were 0.85, 0.85, and 0.84 for crafting for meaning and 0.80, 0.90, and 0.89 for crafting for affiliation, respectively in Japan.

Vitality was measured with four items from the subjective vitality scale by Ryan and Frederick (Ryan and Frederick, 1997; Bostic et al., 2000) at all three time points. An example item is “Over the past month, I felt alive and vital.” Answers could range from 1 (very rarely or never) to 5 (very often or all the time). Cronbach's alphas from T1 to T3 for vitality were 0.93 at all three time points in Finland, and 0.94, 0.96, and 0.93 in Japan.

## Results

To confirm that OJC for meaning and for affiliation and vitality are distinct constructs, we conducted a multigroup confirmatory factor analysis of these three scales at T1. The fit of the three-factor model was acceptable (χ^2^ (74) = 233.03, *p* < 0.001, CFI = 0.96, TLI = 0.95, RMSEA = 0.08, SRMR = 0.05), and all factor loadings were significant (loadings 0.77–0.94, *p* < 0.001). These results were also similar for the T2 and T3 measurements. Finnish employees engaged in OJC for meaning and for affiliation more than Japanese employees at all time points (*t*_(515−715)_ = 10.77–13.87, *p* < 0.001) (Table 1). OJC for meaning was positively correlated to vitality at the within-person level among both Finnish and Japanese employees. OJC for affiliation positively correlated to vitality at the within-person level among Finnish, but not among Japanese employees. Demographic characteristics were modestly related to OJC in zero-order correlations (Table 1).

**Table 1:** ICCs, descriptive statistics (T1), and the intercorrelations between variables (within-person correlations between OJC for meaning, OJC for affiliation and OJC for vitality, and between-person correlations at T1 for all other pairs of variables).

	***ICC***	***M***	***SD***	**1**	**2**	**3**	**4**	**5**	**6**	**7**	**8**
1. Age		48.70/30.86	10.23/ 6.35		−0.09^*^	−0.39^**^	0.57^**^	−0.03	0.10^*^	0.07	0.02
2. Gender		1.86/1.37	0.36/0.50	0.05		0.06	−0.05	−0.03	0.05	0.02	−0.05
3. Education		2.74/3.96	0.91/0.63	−0.06	0.03		−0.33^**^	0.14^**^	−0.02	−0.02	0.05
4. Organizational tenure		14.65/4.85	11.86/4.57	0.49^**^	0.04	0.08		−0.02	0.12^**^	0.08	0.01
5. Working hours		38.88/48.26	4.38/9.79	−0.13^*^	−0.21^**^	0.06	0.02		0.01	0.01	−0.00
6. OJC for meaning	0.59/0.51	3.69/2.61	0.87/1.13	−0.20^**^	0.07	0.09	−0.04	0.01		0.43^**^	0.14^**^
7. OJC for affiliation	0.61/0.54	3.79/2.82	0.82/1.01	−0.06	0.23^**^	0.11	−0.03	0.03	0.45^**^		0.12^**^
8. Vitality	0.67/0.54	3.56/3.36	0.90/1.05	−0.05	0.01	−0.02	−0.01	0.13^*^	0.17^**^	0.07	

*ICCs, means, and standard deviations for Finnish employees before and for Japanese employees after the forward slash. Correlations for Finnish employees above and for Japanese employees below the diagonal. Gender coded as 1 = male, 2 = female. Education (highest qualification obtained) coded as 1 (primary school or lower), 2 (vocational school or college), 3 (vocational institute or bachelor's degree), 4 (master's degree), 5 (higher degree). ICC, intra-class correlation coefficient (ICC1); M, mean; SD, standard deviation; OJC, off-job crafting.*

* *p < 0.05,*

** *p < 0.01*.

### Relationships Between OJC and Vitality

The intraclass correlation coefficients showed that 51–67% of the variation in OJC and vitality could be explained by between-person variation (Table 1). Thus, conducting LGA was appropriate as sufficient variance could be explained by both within- and between-person levels. The univariate latent growth curve model for OJC for meaning (χ^2^ (2) = 0.05, *p* = 0.98, CFI = 1.00, TLI = 1.00, RMSEA = 0.00, SRMR = 0.00) indicated that on an average, scores of OJC for meaning were fairly stable over time for the employees in both countries (FIN slope *M* = 0.01, *SE* = 0.10, p = 0.94; JPN slope *M* = 0.18, *SE* = 0.17, *p* = 0.30). Similarly, the model for OJC for affiliation (χ^2^ (2) = 0.32, *p* = 0.85, CFI = 1.00, TLI = 1.00, RMSEA = 0.00, SRMR = 0.01) indicated that the scores were on average stable over time for the employees in both countries (FIN slope *M* = −0.01, *SE* = 0.10, *p* = 0.94; JPN slope *M* = −0.04, *SE* = 0.10, *p* = 0.71). For vitality (χ^2^ (4) = 11.33, *p* = 0.02, CFI = 0.98, TLI = 0.97, RMSEA = 0.07, SRMR = 0.04), there was a decreasing trend over time for Finnish employees (slope *M* = −0.48, *SE* = 0.18, *p* < 0.01), whereas the average change in vitality over time was non-significant for Japanese employees (slope *M* = 0.55, *SE* = 0.42, *p* = 0.19).

In the next step, we examined the relationships between the development of OJC and the development of vitality, and the baselines of OJC and of vitality with multivariate growth curve models. The model for the relationships between OJC for meaning and vitality (χ^2^ (20) = 58.17, *p* < 0.001, CFI = 0.96, TLI = 0.94, RMSEA = 0.07, SRMR = 0.08) showed that the increase in OJC for meaning over time was positively related to the increase in vitality over time in both the countries (FIN γ = 0.26, *SE* = 0.09, *p* < 0.01; JPN γ = 0.40, *SE* = 0.19, *p* < 0.05). Similarly, the model for the relationships between OJC for affiliation and vitality (χ^2^ (20) = 35.27, *p* < 0.05, CFI = 0.98, TLI = 0.97, RMSEA = 0.04, SRMR = 0.07) showed that the increase in OJC for affiliation over time was also positively related to the increase in vitality over time among Finnish employees (γ = 0.19, *SE* = 0.09, *p* < 0.05) but not among Japanese employees (γ = 0.22, *SE* = 0.14, *p* = 0.10). The initial levels of OJC and vitality were positively correlated in both countries (γ = 0.22–0.51, *SE* = 0.04–0.08, *p* < 0.01). To summarize, H1 was supported, as among Finnish employees the increase in OJC was positively related to the increase in vitality over time. H2 was partially supported, since among Japanese employees only the increase in OJC for meaning (and not OJC for affiliation) was positively related to the increase in vitality over time.

### Contextual Variables and OJC

In the next step, we added each of the contextual variables (gender, age, education, organizational tenure, and working hours) as predictors of the intercepts of OJC for meaning and for affiliation to test hypotheses H3–H6. Model fit remained acceptable for the LGA models for both OJC for meaning and vitality (χ^2^ (70) = 133.47, *p* < 0.001, CFI = 0.94, TLI = 0.93, RMSEA = 0.05, SRMR = 0.07) and OJC for affiliation and vitality (χ^2^ (70) = 106.16, *p* < 0.01, CFI = 0.97, TLI = 0.96, RMSEA = 0.04, SRMR = 0.07). Supporting H3a, age was positively related to OJC for meaning among Finnish employees (γ = 0.13, SE = 0.06, *p* < 0.05), indicating that older Finnish employees engaged in more OJC for meaning at baseline than the younger Finnish employees. Surprisingly, age was negatively related to OJC for meaning among Japanese employees (γ = −0.22, *SE* = 0.09, *p* < 0.05), indicating that younger Japanese employees engaged in more OJC for meaning than the older Japanese employees. Age was not related to OJC for affiliation in either country (γ = −0.08–0.04, *SE* = 0.05–0.07, *p* = 0.30–0.44). Thus, H3a was partially supported, as age was positively related to OJC for meaning among Finnish employees. Contrary to H3b–c, age was related negatively to OJC for meaning among Japanese employees. Gender was related to OJC for affiliation among Japanese employees (γ = 0.25, *SE* = 0.07, *p* < 0.001), indicating that female Japanese employees engaged in OJC for affiliation more than the male Japanese employees. Gender was not related to OJC dimension among Finnish employees (γ = 0.03, *SE* = 0.05, *p* = 0.57) and was also not related to OJC for meaning among Japanese employees (γ = 0.09, *SE* = 0.07, *p* = 0.19). Thus, H4 was partially supported, since female Japanese employees engaged in more OJC for affiliation (but not in more OJC for meaning).

Education was not related to OJC for meaning (γ = 0.10, *SE* = 0.05, *p* = 0.06) or for affiliation (γ = −0.10, *SE* = 0.07, *p* = 0.18) among either Finnish employees or Japanese employees (γ = 0.07–0.10, *SE* = 0.07, *p* = 0.12–0.35). Similarly, tenure (γ = −0.03–0.07, *SE* = 0.06–0.07, *p* = 0.23–0.59) and working hours (γ = 0.02–0.07, *SE* = 0.05–0.07, *p* = 0.31–0.80) were not related to OJC in either country. Thus, H5a–c and H6a–c were not supported.

## Discussion

### Relationships Between OJC and Vitality

We examined the within-person relationships between OJC and vitality over time, expanding research on recovery experiences to focus on the two “forgotten ones,” i.e., recovery experiences of meaning and affiliation (Newman et al., 2014). Supporting hypotheses H1 and H2, the increase in OJC for meaning was positively related to the increase in vitality among both Finnish and Japanese employees, whereas the increase in OJC for affiliation was positively related to the increase in vitality among Finnish, but not among Japanese employees. These results are in line with the DRAMMA model, indicating that aiming to gain experiences of meaning and affiliation is a beneficial strategy to increase well-being. Thus, meaning and affiliation complement the four recovery experiences measured by the REQ as off-job life well-being enhancers (Sonnentag and Fritz, 2007; Newman et al., 2014). Our results also lend indirect support to the integrative needs model of crafting, which suggests that successful OJC efforts lead to higher well-being through satisfying psychological needs, such as meaning and affiliation (de Bloom et al., 2020). Increasing OJC for meaning and for affiliation presumably provides the opportunity for need satisfaction (i.e., experiences of meaning and affiliation), which can be further utilized as personal resources that increase energetic activation (Quinn et al., 2012; Halbesleben et al., 2014). Thus, our results show that shaping off-job life to include more opportunities for experiencing meaning and affiliation can bring increased vitality over time. The null results found for the relationship between the development of OJC for affiliation and vitality among Japanese employees may be due to the smaller sample size in Japan compared with the Finnish sample and the different professions in the two samples, and may also reflect the marked masculinity of Japanese culture (Hofstede et al., 2010). In masculine cultures such as Japan, relatedness and particularly displays of affection between adults are seen as less important aspects of social relationships than in feminine cultures (Hofstede et al., 2010). Thus, the positive effects of OJC for affiliation, such as sharing emotions with significant others, may take more time to unfold in masculine than in feminine cultures (see also Mitchell and James, 2001). To summarize, the results for the relationships between OJC and vitality lend robust support to the positive longitudinal (within-person) associations between OJC for meaning and vitality, and OJC for affiliation and vitality, highlighting that OJC is a beneficial strategy for employees to increase their positive energy.

### Contextual Variables and OJC

Our results demonstrated that Finnish employees consistently engaged in more OJC for meaning and for affiliation than Japanese employees. Leisure is a widely available and autonomy-supporting life domain in Finland (Wang and Wong, 2014), whereas the long hours spent working, socializing with colleagues and clients, and commuting limit the availability of leisure time for Japanese employees (Nemoto, 2013; Statistics Bureau of Japan, 2016). Thus, Finnish employees probably experience more opportunities for crafting their off-job lives than do Japanese employees.

Partially supporting H3a, our results showed that older Finnish employees engaged more in OJC for meaning (but not in OJC for affiliation) than younger Finnish employees at baseline. This finding is in line with studies which show that motives for emotional meaning become more important to people as they age (e.g., Carstensen et al., 1999). Through OJC for meaning, older Finnish employees may seek to make the best out of the more limited time they have left in life (Fung, 2013). However, contrary to H3b and H3c, younger Japanese employees engaged more in OJC for meaning (but not in OJC for affiliation) than older Japanese employees. Thus, it seems that shaping off-job life to experience a sense of purpose is more popular among older employees in Finland, whereas in Japan it is more common among younger employees. This discrepancy in the relationships may be partly explained by recent transitions among the younger generations in Japan. With greatly reduced opportunities for lifelong employment due to the Japanese economic recession in recent decades, Japanese students, in order to attain sustainable future life perspectives for themselves, are increasingly seeking meaning beyond having a stable career path (Kawai and Moran, 2017). This process continues to early working life (Kawai and Moran, 2017). Thus, through OJC for meaning, younger Japanese employees can seek to compensate, through their off-job lives, for the uncertainty caused by uncertain career prospects.

Our results provided partial support for H4, in that female Japanese employees engaged in more OJC for affiliation (but not OJC for meaning) than the male Japanese employees. The results support the notion that the relatively traditional gender roles in the masculine Japanese culture make OJC in the home domain more accessible (and perhaps also more necessary) for Japanese women than for men (Hofstede et al., 2010; Nemoto, 2013). In other words, Japanese women are more likely to craft for and seek affiliation in their off-job lives than men, because of two reasons, namely, they spend more time at home, having more opportunities to craft their off-job life, and also because they may experience a stronger need to seek relatedness in the home domain than men, who have more social contacts at work (Nemoto, 2013; Peltokorpi, 2013). Furthermore, in masculine cultures seeking for relatedness and emotional connection is often seen as a behavior more appropriate to women than men (Hofstede et al., 2010). This may further increase the gender differences in engaging in OJC for affiliation, which may explain why this relationship was found especially for OJC for affiliation in Japan and not for OJC for meaning. In addition, female Japanese employees also experience significantly more stress in interpersonal relationships at work (e.g., difficult or demanding relationships with colleagues or clients) than do male employees, which may increase their need to seek social connection in off-job life (Shimazu, 2020). On the other hand, the feminine values and more widely-available leisure time for both genders in Finland can explain why no gender differences were found in OJC among Finnish employees.

For human capital (education and organizational tenure), even though tenure was positively related to OJC for meaning among Finnish employees in the zero-order correlations, this relationship became non-significant in the models with all contextual variables as predictors of OJC. Since no other relationships emerged between human capital and OJC for meaning or OJC for affiliation, H5a–c were not supported. It may be that the skills employees acquire through education and organizational tenure are not easily translatable to the context of off-job lives.

Similarly, H6a–c were not supported as no relationships were found between working hours and OJC for meaning or OJC for affiliation. It seems that not only Finnish employees, who experience more opportunities to shape their working hours to match with their needs (Härmä, 2006), but also Japanese employees engage in OJC independent of their weekly hours worked, although to a lesser extent (as evident in the lower averages for OJC). It is possible that the relationship between working hours and OJC is more complex than the direct linear relationships tested for in this study. For example, there may be boundary conditions which could explain why working long hours does not reduce OJC for some employees (e.g., if the individual is able to take holidays when needed, or has a personality trait, such as openness to experiences, that could help them in finding opportunities for engaging in OJC even when working long hours).

### Limitations and Suggestions for Future Research

This study has three limitations. First, although we used an identical study procedure among both Finnish and Japanese employees, the samples of Finnish and Japanese employees differed in terms of the professions (e.g., health care in Finland and information technology in Japan) and the distributions of contextual variables (e.g., in terms of age and gender). This was related to the existing company contacts in the two countries and the recruitment process. It is possible that OJC efforts of employees in some professions (e.g., in health care) are more effective in terms of increasing vitality than those of employees in other professions (e.g., in IT). Future studies on OJC could build on this research to investigate OJC in different countries with more homogenous samples. Second, while we present findings between the relationships of OJC and vitality, we examined only this single well-being indicator as an outcome of OJC. For a more comprehensive understanding of the relationship between OJC and well-being, it would be important to examine the role of OJC for other well-being indicators, such as personal growth, self-actualization, or burnout. Third, this study captured only longer-term variations in OJC and vitality (due to the 3-month time lags), leaving potential daily or weekly fluctuations unaccounted for. Diary studies testing the relationships between OJC and well-being would be useful to examine whether the within-person relationships found in this study also exist at the daily or weekly level (e.g., whether individuals who engage in OJC daily experience higher daily well-being). Moreover, qualitative studies would be helpful to gain more insight into what employees specifically do when they engage in OJC for meaning and for affiliation.

### Practical Implications

Even though studies on OJC have so far focused mainly on employees, the concept of OJC is relevant not only for employees, but also, for example, for hobbyists, students, unemployed, and retired individuals, who can also proactively shape their off-job life domains such as leisure and childcare to experience meaning and affiliation. Moreover, the results of this study are encouraging for off-job well-being interventions, which could benefit from a focus on OJC for meaning and for affiliation to help individuals foster their vitality and mental well-being in general as well as job satisfaction (see Sirgy et al., 2020 regarding spillover between life domains). Such interventions could use evidence-based techniques such as writing about values and purposeful goals (Schippers and Ziegler, 2019) or strengths spotting (Kosenkranius et al., 2020). Finally, the disparate results between Finnish and Japanese employees concerning the relationships between contextual variables and OJC provide the first empirical evidence that these relationships are affected by the cultural context, which can promote or hinder OJC. Taking into account the role that culture plays in how individuals shape their off-job life experiences is vital for building more crossculturally sensitive leisure programs and interventions (Edginton et al., 2017).

### Conclusions

In this longitudinal study among Finnish and Japanese employees, we focused on OJC for meaning and OJC for affiliation as predictors of vitality. We moreover studied the relationships of contextual variables such as age and gender with OJC. OJC for meaning and for affiliation were consistently related to vitality in both Finland and Japan longitudinally at the within-person level, the only exception being OJC for affiliation, which was not related to vitality among Japanese employees. To conclude, OJC is beneficial for increasing vitality among Finnish and Japanese employees. The differing relationships between contextual variables and OJC found in the two countries provide the first evidence on how both the cultural and demographic context can affect how people shape their off-job lives to enhance their well-being. However, more research is needed to achieve a better understanding of the role crafting contexts play for OJC.

## Data Availability Statement

The raw data supporting the conclusions of this article will be made available by the authors, without undue reservation.

## Ethics Statement

Ethical review and approval was not required for the study on human participants in accordance with the local legislation and institutional requirements. The patients/participants provided their written informed consent to participate in this study.

## Author Contributions

MKu, AS, and JdB contributed to the conception and design of the study. MKu, AS, HT, and JdB collected the data. MKu performed the statistical analyses. MKu, OW, and JdB wrote the first draft of the manuscript. All authors contributed to, read, and approved the submitted version.

## Conflict of Interest

The authors declare that the research was conducted in the absence of any commercial or financial relationships that could be construed as a potential conflict of interest.

## Publisher's Note

All claims expressed in this article are solely those of the authors and do not necessarily represent those of their affiliated organizations, or those of the publisher, the editors and the reviewers. Any product that may be evaluated in this article, or claim that may be made by its manufacturer, is not guaranteed or endorsed by the publisher.
