# Supplementary material for: The Forgotten Ones: Crafting for Meaning and for Affiliation in the Context of Finnish and Japanese Employees' Off-Job Lives
Source: Front Psychol. 2021 Aug 30;12:682479. doi: 10.3389/fpsyg.2021.682479 (PMC8435721; doi:10.3389/fpsyg.2021.682479)
Supplement: Supplementary file 1 [file Data_Sheet_1.PDF]

Supplementary Material

1 Mplus syntax

Mplus syntax for the final multivariate growth curve model for off-job crafting for meaning and vitality. Contextual variables (age, gender, education, organizational tenure, working hours) were added to the model in a second step.

```
DATA:      FILE = Mplusdata.dat;

VARIABLE:   NAMES = Culture AgeYears
              Sex Education WorkHours EmployerYears
              Vital_MEAN_t1
              OJC_Mea_MEAN_t1 OJC_Aff_MEAN_t1
              Vital_MEAN_t2 OJC_Mea_MEAN_t2 OJC_Aff_MEAN_t2
              Vital_MEAN_t3 OJC_Mea_MEAN_t3 OJC_Aff_MEAN_t3;
              !OJC_Mea = off-job crafting for meaning, OJC_Aff = off-job crafting for affiliation

         USEVAR = AgeYears Sex Education
             WorkHours EmployerYears
             Vital_MEAN_t1
             OJC_Mea_MEAN_t1
             Vital_MEAN_t2 OJC_Mea_MEAN_t2
             Vital_MEAN_t3
             OJC_Mea_MEAN_t3;

         GROUPING IS Culture (1=FIN 2=JPN); !Multi-group analysis

         MISSING = ALL(-999);

ANALYSIS:   ESTIMATOR = MLR;

MODEL:     INT1 SLO1 | Vital_MEAN_t1@0 Vital_MEAN_t2@1 Vital_MEAN_t3@2;
              !Intercept and rate of change vitality
         INT2 SLO2 | OJC_Mea_MEAN_t1@0 OJC_Mea_MEAN_t2@1 OJC_Mea_MEAN_t3@2;
              !Intercept and rate of change OJC for meaning
         [Vital_MEAN_t1@0 Vital_MEAN_t2@0 Vital_MEAN_t3@0 INT1 SLO1];
         [OJC_Mea_MEAN_t1@0 OJC_Mea_MEAN_t2@0 OJC_Mea_MEAN_t3@0 INT2 SLO2];
         SLO1@0.1;
         INT1 WITH SLO1; !Covariance between the intercept and the rate of change
         INT2 WITH SLO2;
         INT2 ON AgeYears Sex Education WorkHours
         EmployerYears; !Contextual variables predicting intercept of OJC for meaning
         INT1 ON INT2; !Intercept of OJC for meaning predicting intercept of vitality
         SLO1 ON SLO2; !Rate of change of OJC for meaning predicting rate of change of vitality
```

```
OUTPUT:    MODINDICES(3.84);
           STDYX RESIDUAL;
```

Mplus syntax for the final multivariate growth curve model for off-job crafting for affiliation and vitality. Contextual variables (age, gender, education, organizational tenure, working hours) were added to the model in a second step.

```
DATA:      FILE = Mplusdata.dat;

VARIABLE:  NAMES = Culture AgeYears
                 Sex Education WorkHours EmployerYears
                 Vital_MEAN_t1
                 OJC_Mea_MEAN_t1 OJC_Aff_MEAN_t1
                 Vital_MEAN_t2 OJC_Mea_MEAN_t2 OJC_Aff_MEAN_t2
                 Vital_MEAN_t3 OJC_Mea_MEAN_t3 OJC_Aff_MEAN_t3;
                 !OJC_Mea = off-job crafting for meaning, OJC_Aff = off-job crafting for affiliation

           USEVAR = AgeYears Sex Education
                WorkHours EmployerYears
                Vital_MEAN_t1
                OJC_Aff_MEAN_t1
                Vital_MEAN_t2 OJC_ Aff _MEAN_t2
                Vital_MEAN_t3
                OJC_ Aff _MEAN_t3;

           GROUPING IS Culture (1=FIN 2=JPN); !Multi-group analysis

           MISSING = ALL(-999);

ANALYSIS:  ESTIMATOR = MLR;

MODEL:     INT1 SLO1 | Vital_MEAN_t1@0 Vital_MEAN_t2@1 Vital_MEAN_t3@2;
                 !Intercept and rate of change vitality
           INT2 SLO2 | OJC_Aff _MEAN_t1@0 OJC_Aff _MEAN_t2@1 OJC_ Aff_MEAN_t3@2;
                 !Intercept and rate of change OJC for affiliation
           [Vital_MEAN_t1@0 Vital_MEAN_t2@0 Vital_MEAN_t3@0 INT1 SLO1];
           [OJC_ Aff_MEAN_t1@0 OJC_ Aff_MEAN_t2@0 OJC_ Aff_MEAN_t3@0 INT2 SLO2];
           SLO1@0.1;
           INT1 WITH SLO1; !Covariance between the intercept and the rate of change
           INT2 WITH SLO2;
           INT2 ON AgeYears Sex Education WorkHours
           EmployerYears; !Contextual variables predicting intercept of OJC for affiliation
           INT1 ON INT2; !Intercept of OJC for affiliation predicting intercept of vitality
           SLO1 ON SLO2; !Rate of change of OJC for affiliation predicting rate of change of vitality
```

```
OUTPUT:     MODINDICES(3.84);
            STDYX RESIDUAL;
```
